# ADHD subtype-specific cognitive correlates and association with self-esteem: a quantitative difference

**DOI:** 10.1186/s12888-020-02887-4

**Published:** 2020-10-12

**Authors:** Parviz Molavi, Mehriar Nadermohammadi, Habibeh Salvat Ghojehbeiglou, Carmelo M. Vicario, Michael A. Nitsche, Mohammad Ali Salehinejad

**Affiliations:** 1 Department of Psychiatry, Fatemi Hospital, School of Medicine, Ardabil University of Medical Sciences, Ardabil, Iran; 2 Department of Scienze Cognitive della Formazione e degli Studi Culturali, University of Messina, Messina, Italy; 3 Department of Psychology and Neurosciences, Leibniz Research Centre for Working Environment and Human Factors, Dortmund, Germany; 4 International Graduate School of Neuroscience, Ruhr-University Bochum, Bochum, Germany; 5 Institute for Cognitive and Brain Sciences, Shahid Beheshti University, Tehran, Iran

**Keywords:** ADHD, ADHD subtype, Cognition, WISC-IV, Working memory, Self-esteem, Personalized medicine

## Abstract

**Background:**

Attention-deficit hyperactivity disorder (ADHD) is a major neurodevelopmental disorder with heterogeneous symptoms, subtypes, and cognitive deficits. Cognitive deficits are central to ADHD pathophysiology and one potential source of heterogeneity in ADHD. Subtype-specific cognitive correlates are not, however, well-studied. We explored cognitive correlates of ADHD *subtypes* based on the Wechsler Intelligence Scale for Children (WISC-IV) scores. We also assessed subtype-specific self-esteem rating in ADHD subtypes and explored its association with cognitive correlates.

**Methods:**

One hundred thirty-nine children with ADHD (80.6% boy, 19.4% girl) were categorized into the predominantly “hyperactive (ADHD-H)”, “inattentive (ADHD-I)” and “combined (ADHD-C)” subtype based on their symptoms and scores on the Kiddie Schedule for Affective Disorders and Schizophrenia (K-SADS-PL) and Conners Parent-Rating Scale (CPRS-RS). They were then individually administrated the WISC-IV and completed a self-esteem inventory. Group differences in the WISC-IV indices and their predictability in discriminating ADHD subtypes were analyzed.

**Results:**

We found a *quantitative* differentiation of cognitive abilities among ADHD subtypes with “working memory” as the most compromised cognitive domain. ADHD-I had the poorest cognitive profile while ADHD-H scored highest in all cognitive domains. Importantly, cognitive abilities were negatively correlated with inattention and positively correlated with hyperactive symptoms. Moreover, self-esteem ratings were positively correlated with the cognitive domains and were rated differently based on the subtypes. ADHD-H, with the highest cognitive strength, reported the highest level of self-esteem among all subtypes.

**Conclusions:**

ADHD subtype-specific symptoms, cognitive deficits, and self-esteem problems should be considered for precise diagnosis and effective and personalized treatment in ADHD in light of further supporting evidence and assessments. Cognitive interventions might be more compatible with and effective in inattentive and combined subtypes of ADHD. Working memory improving-based interventions can benefit all ADHD subtypes. A supportive educational system in school and providing adjunct supportive interventions should be considered for children with ADHD as well.

## Background

Attention-deficit hyperactivity disorder (ADHD) is a major neurodevelopmental disorder with *heterogeneous* symptoms, subtypes and treatment response. A precise description of the pathophysiology underlying ADHD is difficult due to its neuropsychological heterogeneity [1] and substantial overlap between ADHD and typically developing children [2]. Cognitive deficits, especially executive dysfunctions, are central to ADHD psychopathology [3] and among the primary treatment targets by pharmacological [4] and novel treatment approaches [5, 6]. These cognitive deficits are also heterogeneous in ADHD and thus individual differences in cognitive profile should be considered as well [7]. One aspect of heterogeneity in ADHD symptoms and cognitive deficits is its subtypes [8, 9] which includes a predominantly hyperactive (ADHD-H), inattentive (ADHD-I), and combined (ADHD-C) subtype. These subtypes are discerned from each other by the presence of specific symptoms [10], however, little is known about neuro-functional and cognitive differentiation of ADHD subtypes [11].

Furthermore, it is still elusive whether a specific subtype is critical to consider when examining treatment effects. For example, a recent study suggests that the effectiveness of neurofeedback treatment on the executive functioning of children with ADHD is subtype-specific [12]. ADHD-I showed improved performance on the execution of an action in an experimental Go/NoGo task while the ADHD-C showed improved ability to withhold a prepotent response tendency in NoGo trials. Recent reviews of non-invasive brain stimulation studies in ADHD also suggests that the efficacy of the treatment could be different in ADHD subtypes [5, 6]. Identifying subtype-specific profiles in ADHD, especially cognitive profile, is thus a timely and important topic in the field and is in line with recent findings from neuroimaging studies indicating subtype-specific pattern of activity in different brain regions [13, 14].

Previous studies showed that the Wechsler Intelligence Scale for Children (WISC) provides a relatively comprehensive profile of cognitive strengths and weaknesses and is commonly used for cognitive evaluation in the clinical pediatric population. Application of WISC in ADHD also confirms that it reliably differentiates between ADHD patients and healthy controls [15, 16]. Moreover, it can provide knowledge about specific cognitive strengths and weaknesses in ADHD [17–21], used in designing therapeutic and educational interventions, and employed as a diagnostic marker in children with ADHD [17, 22–24]. However, the cognitive strengths and weaknesses of ADHD *subtypes* are not adequately explored in the previous studies in ADHD while several neuropsychological distinctions in ADHD subtypes (especially ADHD-I vs ADHD-C) have been identified [25].

Similar to subtype-specific cognitive deficits, self-esteem and social functioning in ADHD subtypes are not well-studied and the available studies are even more limited. Lower ratings of self-esteem in ADHD patients compared to the healthy control, regardless of ADHD subtypes, have been reported in previous studies [26–28]. These studies also showed that the treatment of ADHD symptoms was associated with the improvement of self-esteem scores [27] suggesting that self-esteem and ADHD symptoms (including cognitive deficits) are relevant for treatment efficacy. Nevertheless, subtypes-specific ratings of self-esteem and its association with cognitive deficits require further investigation.

The purpose of the present study was, therefore, to explore cognitive correlates of ADHD subtypes based on the WISC-IV scores in a relatively large sample size (*n* = 139). We also performed a discriminative analysis to evaluate if the cognitive profile of each ADHD subtype can predict group membership. We further assessed the level of self-esteem in each ADHD subtypes to determine any associations between subtype-specific cognitive correlates and self-esteem ratings in ADHD.

## Methods

### Participants

One hundred thirty-nine children with ADHD (80.6% boy, 19.4% girl, mean age = 8.20 ± 2.50), referred to the Fatemi Hospital at Ardabil University of Medical Sciences, were included in this study. The data was collected in the child psychiatry division of the hospital which is dedicated to the diagnosis and treatment of child and adolescence psychiatric disorders, including neurodevelopmental disorders (e.g. ADHD, autism, learning disabilities). Participants’ recruitment took place from mid-2017 until the end of 2019. The inclusion criteria were: (1) ADHD diagnosis according to the DMS-5 criteria by a licensed psychiatrist and a child psychologist, (2) moderate to severe score in the parent or teacher version of the Conners’ Parent Rating Scale (CPRS) in addition to an independent diagnosis made by psychiatrist/psychologist, (3) being 6–15 years old, (4) and no current and history of psychiatric, neurodevelopmental disorders and chronic physical illness. All participants were on medication treatment when recruited for the experiment however, there were prevented from the medication 24 h before testing to ensure that WISC-IV performance was not affected by medication as suggested by previous studies [21, 29, 30]. The study was performed according to the latest version of the Declaration of Helsinki and approved by the ethics committee of the local University. Participants’ parents were instructed about experimental procedures and gave their written informed consent. Demographic information is summarized in Table 1.

**Table 1:** Demographic information

Demographic information					
Variable			value	***p***	Group difference^*^
Sample size (*n*)			139		
	subtype	ADHD-H (%)	71 (51.1%)		
		ADHD-I (%)	35 (25.2%)		
		ADHD-C (%)	33 (23.7)		
Age	Mean (SD)	Total	8.20 (2.50)	0.001	ADHD-H < ADHD-I
		ADHD-H	7.45 (2.45)		
		ADHD-I	9.54 (2.29)		
		ADHD-C	8.42 (2.26)		
Child order among siblings	1st	ADHD-H/I/C	41 / 18 / 27		
	2nd	ADHD-H/I/C	22 / 8 / 5		
	3rd	ADHD-H/I/C	8 / 7 / 0		
	4th	ADHD-H/I/C	0 / 1 / 0		
	5th and higher	ADHD-H/I/C	0 / 1 / 1		
Gender (*n*)	Male (female)		112 (27)		
CPRS-RS	Inattaention	ADHD-H (SD)	26.97 (4.81)	0.001	ADHD-I > ADHD-H
		ADHD-I (SD)	40.60 (4.37)		ADHD-C > ADHD-H
		ADHD-C (SD)	42.72 (4.48)		
	Hyperactivity	ADHD-H (SD)	33.11 (4.37)	0.001	ADHD-H > ADHD-I
		ADHD-I (SD)	23.42 (4.69)		ADHD-C > ADHD-I
		ADHD-C (SD)	40.66 (4.18)		
	ADHD-index	ADHD-H (SD)	59.14 (4.54)	0.001	ADHD-C > ADHD-I
		ADHD-I (SD)	64.42 (6.26)		ADHD-C > ADHD-H
		ADHD-C (SD)	83.30 (7.40)		

Note: *SD* Standard Deviation, *ADHD-H* Predominantly hyperactive, *ADHD-I* Predominantly inattentive, *ADHD-C* Combined, *CPRS-RS* Conners Parent Rating Scale-Revised: Short form. Between-group differences in demographic continuous variables were explored by F-test. * indicates that only significant group difference between ADHD subtypes are presented.

### Measures

#### Kiddie schedule for affective disorders and schizophrenia (K-SADS-PL)

The K-SADS-PL (3) is a semi-structured interview for assessing psychiatric diagnoses in children and adolescents from 6 to 18 years old. It assesses the present, history of psychiatric disorders, and the severity of symptoms based on the DSM-IV. The K-SADS-PL-P is especially sensitive at diagnosing patients with ADHD, major depressive disorder, bipolar disorder, general anxiety disorder, post-traumatic stress disorder, and substance use disorder. The test-retest reliability and kappa coefficients are reported excellent for the present and lifetime diagnosis of major psychiatric disorders. A native-language version of the scale used in this study with good-to-excellent concurrent validity in diagnosing current major disorders [31]. The Kappa agreements for most diagnoses are higher than 0.4 and the test-retest reliability is 0.87.

#### Conners’ parent rating scale-revised: short version (CPRS-RS)

The revised version of Conners’ Rating Scale [32] has three forms; parent, teacher and self-report form and each form has a short and long version. The CPRS-RS used in this study contains 27 items and measures symptoms in four subscales: 1) oppositional subscale, 2) inattention, 3) hyperactive/impulsive, and 4) ADHD Index. Each item is presented in a 4-point Likert scale ranging from Not True at All (1) to Very Much True (4). The cut-off point for clinical diagnosis of ADHD is reported differently and we used scores higher than 59 based on CPRS-RS guidelines which is indicative of “higher average score”. The items are based on DSM diagnostic criteria for ADHD. Psychometric properties of the CPRS-RS are reported adequate as demonstrated by good internal reliability coefficients, high test-retest reliability and effective discriminative power [33]. The psychometric properties of the native version of the CPRS are reported good and reliable and demonstrated to be useful in discriminating children with ADHD from typically developing individuals [34].

#### Wechsler intelligence scale for children (WISC-IV)

The WISC-IV is an individually administered test battery that assesses intelligence in school-aged children (from 6 to 16 years of age) [35, 36]. The 4th edition included 10 subtests yielding to four index scores that combine into one FSIQ. The index scores include (1) Verbal Comprehension Index (VCI), (2) Perceptual Reasoning Index (PRI), (3) Work Memory Index (WMI), and (4) Processing Speed Index (PSI). Analyzing WISC-IV profiles are suggested as useful differential diagnosis tools, particularly in distinguishing between “real ADHD” and pseudo-ADHD [37, 38].

#### Coopersmith self-esteem (CSE) inventory

The 58-items CSE with high reliability of 0.88 and validity [39] was used to measure the level of self-esteem in participants. CSE is one of the most commonly-used measure of self-esteem in healthy and clinical populations [40]. In CSE, self-esteem total score ranges from 0 to 50. Scores higher than 25 indicate high levels and scores lower than 25 indicate a low level of total self-esteem score. The total self-esteem score is the compound score consisting of four subscales in including global self-esteem, social self-esteem, family self-esteem, and educational/professional self-esteem. These subscales have a separate score and combination of them represents total self-esteem score. The CSE thus measures self-esteem as a global score but it yields a specific score for each subscale as well. The CSE has reliability and validity [41] and the Cronbach’s α coefficient and split-half reliability are reported to be 0.83 and 0.84, respectively [42, 43].

### Procedure

139 children in this study were consecutive referrals to our child psychiatry hospital who were diagnosed with ADHD. After we received institutional review board approval, children with ADHD and their parents were interviewed by a psychiatrist based on the CPRS-RS and K-SADS-PL and were categorized into ADHD-I, ADHD-H, and ADHD-C accordingly. Twenty-four hours before the testing day, the patients that were on medication stopped taking the medication. They were then administrated the WISC-IV and completed the self-esteem inventory. All tests were conducted in the same testing room. The order of tests was randomized.

### Statistical analysis

We used IBM Statistical Package for the Social Sciences (SPSS) for Windows, version 24 (SPSS Inc., Chicago, IL, USA) for data analysis. A one-way analysis of variance (ANOVA) and Bonferroni-corrected t-tests were applied to examine group differences for the major WISC-IV indices, including the VCI, PRI, WMI, PSI, and the full-scale intelligence quotient (FSIQ). It is of note that the potential covariate effect of age was controlled as FSIQ and indices scores are estimated by age-scaled scores and thus age was not entered a covariate in the analysis. A separate similar ANOVA was conducted on the rating of self-esteem domains. Our data met the ANOVA linear assumptions and Leven’s test was used to examine the homogeneity of variances. Additionally, we performed a discriminant analysis to explore whether WISC-IV indices scores, as predictor variables, can predict grouping of ADHD patients into ADHD-I, ADHD-H, and ADHD-C subtypes. Correlational analyses between the outcome measures were calculated using the Pearson correlation (two-tailed). A significance level of *p* < .05 was used for all statistical comparisons.

## Results

### Cognitive profile differences in ADHD subtypes

**Fig. 1:**
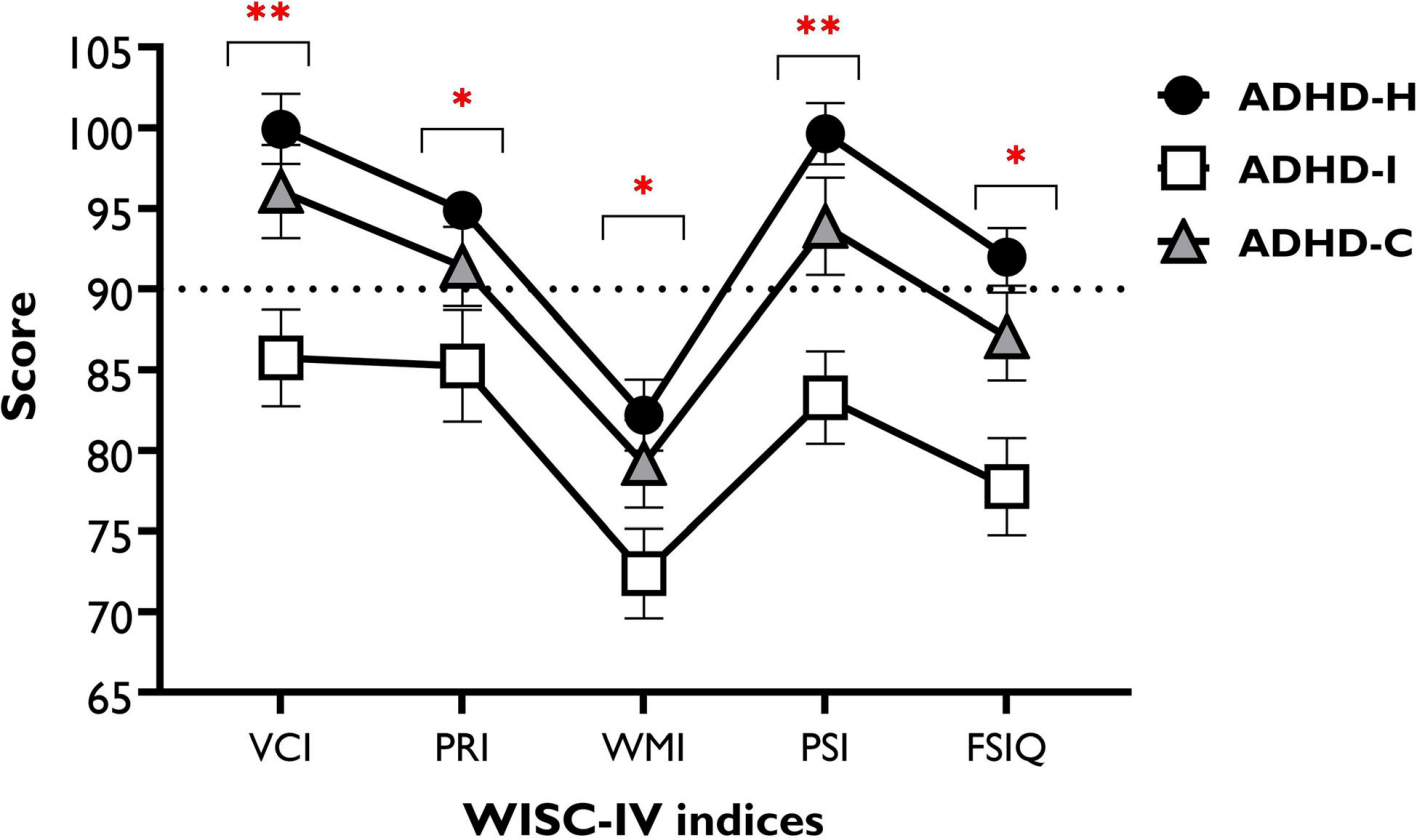
Mean of the WISC index scores and FSIQ in ADHD subtypes (*n* = 139) *Note*: ADHD-H: predominantly hyperactive; ADHD-I: predominantly inattentive; ADHD-C: combined; WISC: Wechsler Intelligence Scale for Children; VCI = Verbal Comprehension Index; PRI = Perceptual Reasoning Index; WMI = Working Memory Index; PSI = Processing Speed Index; FSIQ = Full-Scale Intelligence Quotient; * = indicates significant difference of ADHD-I vs ADHD-H; ** = indicates significant difference of the ADHD-I group vs both, ADHD-H and ADHD-C groups. Post-hoc comparisons were conducted using the Bonferroni-corrected post hoc t-tests. All error bars represent standard error of mean (SEM)

The results of ANOVA showed significant differences between ADHD subtypes that were revealed in the FSIQ and all indices of VCI, PRI, WMI, and PSI (Fig. 1, Table 2). Bonferroni-corrected post hoc t-tests revealed that ADHD-I, compared to the ADHD-H, scored lower in the FSIQ (*t* = 4.21, *p* < 0.001) and all indices of VCI (*t* = 4.20, *p* < 0.001), PRI (*t* = 2.85, *p* = 0.013), WMI (*t* = 2.90, *p* = 0.011), and PSI (*t* = 4.84, *p* < 0.001). Moreover, ADHD-I scores in the VCI (*t* = 2.59, *p* = 0.028) and PSI (*t* = 2.67, *p* = 0.023) indices were significantly lower than ADHD-C. No significant difference between ADHD-H vs ADHD-C was found in any of the indices. In sum, WMI was the weakest (regardless of ADHD subtype), VCI was the strongest index, and FSIQ score was 3 points lower than 90 (Fig. 1).

**Table 2:** ANOVA results for the group differences in the WISC indices and self-esteem rating

WISC-IV							
**variable**	**indices**	**Group Mean (SD)**					
		**ADHD-H**	**ADHD-I**	**ADHD-C**	**F**	***p*-value**	**η**_**p**_^**2**^
**WISC-IV**	VCI	99.94 (18.27)	85.74 (17.72)	96.06 (16.58)	7.52	**<.001**	.10
	PRI	94.90 (9.22)	85.25 (20.47)	91.42 (14.08)	5.58	**.005**	.076
	WMI	82.19 (18.59)	72.37 (16.44)	79.18 (15.57)	3.74	**.026**	.052
	PSI	99.64 (15.83)	83.28 (16.98)	93.90 (17.35)	11.48	**.001**	.144
	FSIQ	92.00 (15.12)	77.77 (17.77)	87.06 (15.63)	9.33	**<.001**	.121
**Self-esteem (CSE)**							
**variable**	**domain**	**Group Mean (SD)**					
		**ADHD-H**	**ADHD-I**	**ADHD-C**	**F**	***p*-value**	**η**_**p**_^**2**^
**CSE**	Global	21.11 (2.18)	18.14 (3.44)	17.06 (3.89)	24.65	**<.001**	.266
	Total	38.52 (5.79)	33.00 (8.18)	32.30 (7.00)	13.18	**<.001**	.162
	Social	6.45 (1.54)	5.57 (2.26)	5.69 (1.64)	3.72	**.027**	.052
	Educational	4.94 (1.88)	3.82 (2.39)	4.12 (2.24)	3.85	**.024**	.054
	Family	5.98 (1.93)	5.45 (1.40)	5.42 (1.75)	1.64	.198	.024

Note: *WISC* Wechsler Intelligence Scale for Children, *ADHD-H* predominantly hyperactive, *ADHD-I* predominantly inattentive, *ADHD-C* Combined, *VCI* Verbal Comprehension Index, *PRI* Perceptual Reasoning Index, *WMI* Working Memory Index, *PSI* Processing Speed Index, *FSIQ* Full-Scale Intelligence Quotient, *CSE* Coopersmith Self-esteem Inventory; Post-hoc comparisons were conducted using the Bonferroni-corrected post-hoc t-tests. All error bars represent s.e.m.; significant results are bolded (*p* < 0.5)

### Discriminant analysis and predictive ability

We used discriminant analysis to see whether subtests scores of the WISC-IV, as predictor variables, can predict grouping of ADHD patients into ADHD-I, ADHD-H, and ADHD-C. Results of the discriminant analysis showed a significant function (*p* = 0.001) that accounted for 90.7% of the discriminative ability of the WISC-IV subtests in predicting ADHD group membership (Chi-square = 113.27, df = 20, *p* = 0.001). The canonical correlation between predictor variables and grouping was R = 0.727. Correct grouping of the function for ADHD-I, ADHD-H, and ADHD-C was 57.1, 84.5, and 45.5% respectively. Moreover, the discriminant function could correctly classify 68.3% of the individuals or identified the group to which the individuals belong (Table 3). Finally, we calculated coefficients of the WISC-IV subtests, which can specify the contribution of each WISC-IV subtests to distinguishing or discriminating ADHD subtypes. The vocabularies (0.508), similarities (0.364) and symbol search (0.258) subscales had the most significant correlation with a discriminant function (Table 3).

**Table 3:** Results of discriminant analysis about predicted group membership

Eigenvalues				Wilks’ Lambda			
Function	Eigenvalue	% of variance	Canonical r	Wilks Lambda	Chi-square	df	Sig
1	1.122	90.7	.727	.423	113.279	20	**.000**
2	.115	9.3	.322	.896	14.370	9	.110
**Groups**		**N (%)**			**Total**		
		ADHD-H	ADHD-I	ADHD-C			
ADHD-H		60 (84.5)	6 (8.5)	5 (7.0)	71 (100.0)		
ADHD-I		2 (5.7)	20 (57.1)	13 (37.1)	35 (100.0)		
ADHD-C		8 (24.2)	10 (30.3)	15 (45.5)	33 (100.0)		

a.68,3% of original grouped cases correctly classified

Note: *ADHD-H* predominantly hyperactive, *ADHD-I* Predominantly inattentive, *ADHD-C* Combined

### Self-esteem rating

**Fig. 2:**
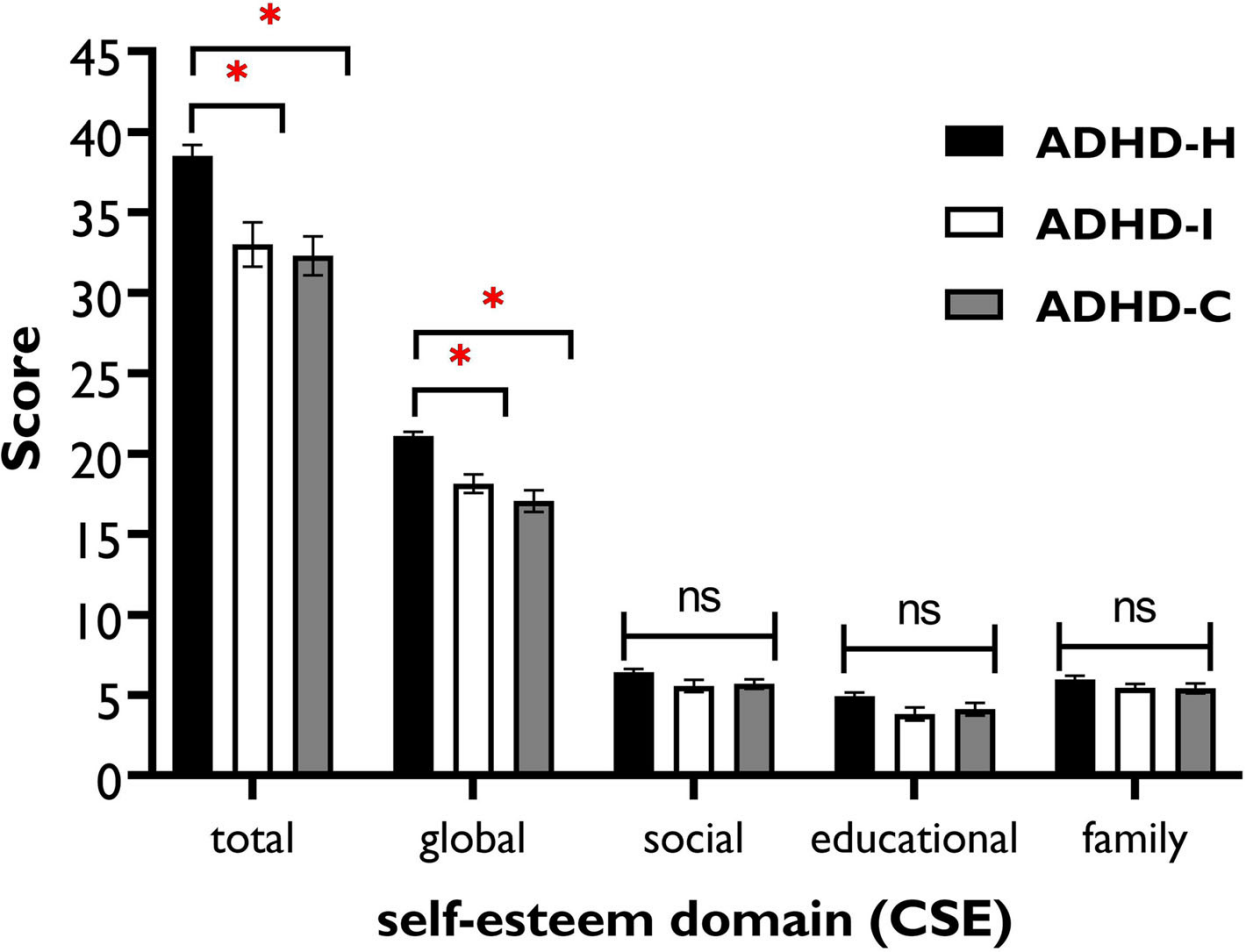
Mean of the self-esteem domains scores in ADHD subtypes (*n* = 139) *Note*: ADHD-H: predominantly hyperactive; ADHD-I: predominantly inattentive; ADHD-C: combined; CSE = Coopersmith self-esteem Inventory; * = indicates significant difference. Post hoc comparisons were conducted using the Bonferroni-corrected post hoc t-tests. All error bars represent standard error of mean (SEM)

Results of the ANOVA showed significant differences between ADHD subtypes in the global and total self-esteem rating and a significant main effect of subtypes was found (Table 2). Bonferroni-corrected post hoc analysis revealed that ADHD-H had a significantly higher *total* self-esteem compared with ADHD-I (*t* = 7.39, *p* < 0.001) and ADHD-C (*t* = 8.16, *p* < 0.001). Similarly, ADHD-H patients had a significantly higher *global* self-esteem compared with ADHD-I (*t* = 3.97, *p* < 0.001) and ADHD-C (*t* = 5.32, *p* < 0.001). No significant differences were found in the subdomains of self-esteem between the groups (Fig. 2).

### Correlational analyses

Cognitive indices of the WICS-IV were positively correlated with ratings of self-esteem indicating that cognitive deficits were associated with lower self-esteem. This positive correlation was observed in all of the WICS-IV indices (VCI, PRI, WMI, PSI, FSIQ) and all of the self-esteem domains except for the family self-esteem (Table 4). Furthermore, we found interesting associations between the WICS-IV indices and attentional vs hyperactivity scores. Cognitive deficits were negatively correlated with attentional symptoms which means that more attentional deficits were associated with poor performance on the WICS-IV indices. In contrast, performance on the WICS-IV indices was positively correlated with hyperactivity/impulsivity symptoms (Table 4). This pattern of association is in line with the quantitative difference between the ADHD-I and ADHD-H with better scores on WICS-IV indices for the latter group. Finally, all of the self-esteem domains, except for the family self-esteem, were negatively correlated with inattention symptoms but not hyperactivity.

**Table 4:** Correlational analyses between WISC-IV indices and CSE self-esteem domains

	Global SE	Total SE	Family SE	Social SE	Educational SE	inattention	hyperactivity	ADHD index
FSIQ	0.520**	0.576**	0.069	0.546**	0.600**	−0.334**	0.182*	−0.156
VCI	0.505**	0.541**	0.008	0.519**	0.580**	−0.256**	0.203*	−0.083
PRI	0.427**	0.463**	0.051	0.453**	0.465**	−0.379**	0.162	−0.216**
WMI	0.468**	0.503**	0.104	0.455**	0.479**	−0.166	0.076	−0.078
PSI	0.391**	0.482**	0.107	0.442**	0.540**	−0.318**	0.189*	−0.129
Global SE	**–**	**–**	**–**	**–**	**–**	−0.473**	−0.025	− 0.415**
Total SE	**–**	**–**	**–**	**–**	**–**	−0.387**	− 0.003	−0.303**
Family SE	**–**	**–**	**–**	**–**	**–**	−0.066	−0.161	− 0.101
Social SE	**–**	**–**	**–**	**–**	**–**	−0.244**	0.018	−0.177**
Educational SE	**–**	**–**	**–**	**–**	**–**	−0.289**	0.137	−0.132

*Note*: *WISC* Wechsler Intelligence Scale for Children, *ADHD-H* Predominantly hyperactive, *ADHD-I* Predominantly inattentive, *ADHD-C* Combined, *VCI* Verbal Comprehension Index, *PRI* Perceptual Reasoning Index, *WMI* Working Memory Index, *PSI* Processing Speed Index, *FSIQ* Full-Scale Intelligence Quotient, *SE* Self-esteem; All analyses are with Pearson correlation; * = correlation is significant at 0.05 (two-tailed); ** = correlation is significant at 0.01 (two-tailed); Significant results are bolded

## Discussion

In the present study, we explored subtype-specific cognitive correlates in 139 children with ADHD based on the WISC-IV. We also assessed self-esteem ratings in ADHD subtypes. Our results show that ADHD-I has the most impaired cognitive profile among all ADHD subtypes and is mostly discriminated with ADHD-H, with the least impaired cognitive functions. Our results further suggest a quantitative differentiation of cognitive profiles among ADHD subtypes with working memory as the most compromised cognitive domain with the lowest value in ADHD-I. Moreover, we found a converging pattern of ADHD subtype-specific differences in self-esteem rating with a significantly higher-rated self-esteem in ADHD-H compared to ADHD-I and ADHD-C.

With regard to subtype-specific cognitive correlates, we found a quantitative differentiation of cognitive profiles among ADHD subtypes regardless of WISC-IV domains in line with previous works [7, 44, 45]. This suggests that all ADHD subtypes display similar cognitive deficits with WM as the most impaired domain but the extent to which ADHD symptoms are close to inattentive vs hyperactive subtype determines the level of impairment. This is in line with the results of Roberts et al. (2017) that found group difference in executive dysfunction of ADHD subtypes based on gradations of EF impairments. The group with poor set-shifting/speed, close to ADHD-I subtype in our study, was the most severely impaired one and the intact task performance group (close to ADHD-H in our study) was relatively unimpaired in executive functioning task performance [7]. This finding is in line with a previous study based on a sample size of 1038 children with ADHD that found those with cognitive subtype (close to ADHD-I in our sample) exhibit information processing deficits (PSI index in our sample) compared to subtypes with more predominately behavioral problems (ADHD-H in our sample) [46]. This study also reported that ADHD subtypes can be described on a continuum of severity which is supported by our findings.

An important aspect of our findings was that WM is the poorest cognitive domain in all ADHD subtypes especially in the ADHD-I. Previous studies using the Wechsler Intelligence Scale in both children and adults with ADHD, regardless of subtype, showed that working memory and processing speed are usually among the most impaired domains in ADHD patients compared to healthy controls [15, 21, 29, 47]. A recent study that comprehensively examined executive functioning heterogeneity in pediatric ADHD using neuropsychological battery also found a positive association between impaired working memory and exhibiting higher ADHD symptoms [48]. Our results are generally in line with these studies by showing WM as the most impaired domain in all *subtypes*. The quantitative pattern observed in cognitive profiles of ADHD subtypes might also suggest that WM is the underlying basic executive function [49] that affects performance on other cognitive domains [50]. More importantly, this is in line with a recently introduced model of ADHD psychopathology, which proposes WM deficits as a major risk factor in ADHD [51] and implies that WM is probably one of the core cognitive deficits in the pathophysiology of all ADHD subtypes, and could be a reasonable target for ADHD treatment. Novel treatment approaches, such as non-invasive brain stimulation that have been promisingly used in ADHD [5, 6] also target WM as one of the core deficits in ADHD.

In addition to the cognitive correlates, we found a subtype-specific pattern in self-esteem ratings with ADHD-H reporting a higher level of global and total self-esteem compared to other subtypes. Previous studies documented social impairment in ADHD and emphasized on the need for further investigation of subtype-specific social deficits in ADHD [52]. In line with this, our results showed that self-esteem ratings of children with ADHD follow the same quantitative pattern of response in cognitive correlates. In other words, those subtypes with more severe cognitive deficits had the lowest level of self-esteem as well which was supported by the negative correlation between the self-esteem domains and cognitive correlates. The association of subtype-specific cognitive correlates and self-esteem rating is novel and not well-studies by previous works but is in line with studies showing an association between self-esteem and cognitive performance [53, 54].

One important point to be noted here is the distribution of ADHD subtypes in our sample. Participants with the ADHD-H subtype constitute the majority of our sample (51.1%) while in other studies, the ratio of ADHD-I and ADHD-C has been relatively reported higher [55, 56]. One potential reason could be that the majority of the sample were boys with ADHD who relatively have a higher ADHD-H subtype ratio than females [57–59] although results have been mixed and some studies show no gender difference between ADHD subtypes. Furthermore, the distribution of ADHD subtype in the Iranian sample does not follow those of western countries [60] and this could be another reason for such a subtype-specific ratio in our sample. Finally, different ratings of symptoms and diagnosis strategy, which can be affected by cultural factors too, could also contribute to higher ADHD-H ratio in our sample.

Taken together, the results of the present study show that there is subtype-specific cognitive profile, measured by WISC-IV, in ADHD confirming a cognitive heterogeneity in ADHD in line with recent evidence [48]. ADHD subtype, if is reliably identified and existed, is an important contributing factor not only to cognitive strength/weakness but also self-esteem ratings. These results have implications for diagnosis precision and personalized treatment in ADHD patients. For instance, cognitive interventions are among the major treatments in ADHD which might be more compatible with and effective in ADHD-I or ADHD-C subtypes due to more severe cognitive weaknesses. Similarly, social interventions and self-esteem can be more effectively addressed in the subtypes with lower self-esteem ratings. The need for the individualized and personalized treatment approach in ADHD is supported more than before from the neurobiological differences of ADHD subtypes [11] and is required due to the heterogeneity of ADHD symptoms.

It is necessary to note that although the focus of this study is on subtype-specific differences in cognitive and self-esteem profiles of children with ADHD, such subtype specification is not confirmed in all studies. Some studies have shown an instability or shift in subtypes over years in children with ADHD [61, 62]. Findings of these studies indicate that ADHD subtypes distinction may not provide a reliable approach for long-term diagnosis and treatment in children with ADHD, especially when the subtype diagnosis is not validated via different measures and assessments. Nevertheless, finding from neuroimaging studies that partially supports a subtype-specific involvement of brain region in ADHD [13, 14] is needed in the future studies along with other neuropsychological measures to validate subtype-specific diagnose in children with ADHD.

The following limitations should be considered. First, we did not have a control group consisting of typically developing children because the purpose of this study was to determine subtype-specific cognitive differences in ADHD. Nevertheless, comparison with typically developing children can also reveal insightful differences of children with ADHD compared to their healthy peers. Second, because we did not intend to focus on gender differences, the number of girls in the study was small and need to be explored in larger samples. Third, the WISC-IV indices might not examine specific aspects of cognition in ADHD and cognitive profile of ADHD subtypes needs to be explored with more specific cognitive measures of executive functions, such as cold vs hot executive functions [63]. Finally, the concept of subtype distinction among children with ADHD is a controversial topic and may not be consistent across the life span which needs to be considered in interpreting the results of this study. These limitations notwithstanding, our ADHD sample were recruited from clinical settings, rather than community, and could have clinical implications.

## Conclusions

We found a subtype-specific quantitative difference in cognitive correlates and self-esteem ratings of children with ADHD which can be considered for precise diagnosis and individualized interventions. Further assessments and neuroimaging findings in support of a subtype-specific distinction are needed. Our findings also support the notion that ADHD is characterized by neurocognitive heterogeneity. Cognitive interventions might be more compatible with and effective in inattentive and combined subtypes of ADHD but working memory improving-based interventions can benefit all subtypes. Association of cognitive performance and self-esteem ratings indicates the importance of educational support system in school for children with ADHD and/or providing adjunct supportive interventions in addition to cognitive ones.

## Data Availability

The datasets used and/or analyzed during the current study are available from the corresponding author on reasonable request.

## Abbreviations

ADHD: Attention-deficit hyperactivity disorder
ADHD-C: Combined
ADHD-H: Predominantly hyperactive
ADHD-I: Predominantly inattentive
CPRS-RS: Conners Parent Rating Scale-Revised Short
DSM-5: Diagnostic and Statistical Manual of Mental Disorders, Fifth Edition
FSIQ: Full-Scale Intelligence Quotient
VCI: Verbal Comprehension Index
PRI: Perceptual Reasoning Index
WMI: Working Memory Index
PSI: Processing Speed Index
CSE: Coopersmith self-esteem Inventory
